# In the Words of Others: ERP Evidence of Speaker‐Specific Phonological Prediction

**DOI:** 10.1111/psyp.70135

**Published:** 2025-09-01

**Authors:** Marco Sala, Francesco Vespignani, Simone Gastaldon, Laura Casalino, Francesca Peressotti

**Affiliations:** ^1^ Department of Developmental Psychology and Socialisation University of Padova Padova Italy; ^2^ Padova Neuroscience Center (PNC), University of Padova Padova Italy

**Keywords:** ERP, foreign‐accent, language comprehension, linguistic prediction, psychophysiology

## Abstract

Prediction models usually assume that highly constraining contexts allow the pre‐activation of phonological information. However, the evidence for phonological prediction is mixed and controversial. In this study, we implement a paradigm that capitalizes on the phonological errors produced by L2 speakers to investigate whether specific phonological predictions are made based on speaker identity. L1 Italian speakers were asked to read sentence fragments, after which a final word was spoken by either an L1‐ or an L2‐accented speaker. The spoken final word could be predictable or not, depending on the sentence context. The identity of the speaker (L1‐ vs. L2‐accented) may or may not be cued by an image of the face of the speaker. Our main analysis indicated that cueing the speaker identity was associated with a larger N400 predictability effect, possibly reflecting an easier processing of predictable words due to phonological pre‐activation. As visual inspection of the waveforms revealed a more complex pattern than initially anticipated, we used Temporal EFA (Exploratory Factor Analysis) to identify and disentangle the ERP components underlying the effect observed. In the L1‐accent condition, predictable words elicited a posterior positivity relative to unpredictable words, possibly reflecting a P3b response, which was more pronounced when the speaker identity was cued. In the L2‐accent condition, cueing the speaker identity was associated with a smaller N1 and a larger P3a response. These results suggest that phonological prediction for L1‐ and L2‐accented speakers likely involves different cognitive processes.

## Impact Statement

Predictive processes operate across multiple levels of language, as shown by research investigating modulations of the N400 amplitude. Despite the central role of phonological information in real‐time language processing, it is debated whether and how phonologically based prediction impacts N400 amplitude and earlier ERP components. The present study advances this understanding by providing novel and compelling evidence for the engagement of phonological prediction during speech comprehension and offering new insights into the physiological responses that underlie this process.

## 1 Introduction

In both oral and written language, the intended meaning of a sentence emerges as more information becomes available. The linearity of the linguistic input suggests that complex meanings are constructed gradually as words are processed: the meaning of each word is retrieved from the continuous sensory input and integrated into an unfolding representation of the preceding context (Altmann and Mirković 2009; Frazier and Rayner 1982). Nevertheless, compelling evidence from different cognitive domains has shown that the human brain predicts incoming information to optimize information processing (Cheung and Bar 2012; Costa et al. 2024; Friston 2005).

Prominent models of language comprehension propose that highly constraining contexts enable the pre‐activation of meaning and form of the expected linguistic input (Altmann and Mirković 2009; Dell and Chang 2014; Kuperberg and Jaeger 2016; Pickering and Gambi 2018; Pickering and Garrod 2007, 2013). Consistent evidence has been provided for the pre‐activation of both semantic (Altmann and Kamide 1999, 2007; Chambers et al. 2002; Federmeier and Kutas 1999; Kamide et al. 2003; Metusalem et al. 2012; Paczynski and Kuperberg 2012) and syntactic (Crocker 2000; Kimball 1975; Levy 2008; Lewis 2000; Staub and Clifton 2006; Traxler 2014; Traxler et al. 1998; van Gompel et al. 2005) information during sentence processing. However, the extent to which comprehenders can predict phonological forms in highly constraining contexts remains a matter of debate, with current models of language comprehension remaining rather underspecified in this regard.

In the event‐related potentials (ERP) literature, most studies investigating whether sentence contexts are used to predict the phonological form of a highly predictable word have focused on the N400 component (DeLong et al. 2005, 2019, 2021; Ito et al. 2016, 2017, 2020; Martin et al. 2013; Nieuwland et al. 2018). The N400 is a negative‐going, centro‐parietally distributed component of the ERP, peaking around 400 ms after word onset and strongly associated with lexical and semantic processing (Kutas and Federmeier 2011; Kutas and Hillyard 1980, 1984). The N400 is typically elicited by open‐class words (Kutas and Van Petten 1988) and is smaller for high‐frequency words and for words that have been semantically primed. Within sentences, the N400 amplitude is highly correlated with an offline measure of the eliciting word's predictability, known as *cloze probability*—the proportion of individuals who continue a sentence fragment with a given word—where higher cloze probability results in a smaller N400 amplitude (Kutas and Federmeier 2011). Two main proposals have been made regarding the cognitive processes underlying the modulation of the N400 amplitude. The *integration view* hypothesizes that facilitatory effects of context arise only when the features of a verbal input have already been accessed through bottom‐up processing, reflecting the degree of (mis)match between the context features and the current word features and/or an easier process of linking the current word with prior context information (Kutas and Federmeier 2011; Van Petten 1993; van Berkum et al. 1999; Van Petten and Luka 2012). The *prediction view* posits that facilitatory effects of context emerge due to the top‐down pre‐activation of upcoming words, resulting in an easier retrieval of the corresponding lexical‐semantic representation from long‐term memory (DeLong et al. 2005; Federmeier and Kutas 1999; Kuperberg 2016; Kutas and Federmeier 2011; Lago et al. 2023). The two views are not incompatible with each other, and some researchers argue that the N400 does not reflect a single process but rather the combined activity of multiple processes, indexing both the pre‐activation of the upcoming linguistic information and its integration in the sentence context (Baggio 2012, 2018; Baggio and Hagoort 2011; Kutas and Federmeier 2011; Newman et al. 2012; Pylkkänen and Marantz 2003). Emerging evidence suggests that these mechanisms may operate in parallel, contributing to similar but distinct subcomponents of the N400 (Nieuwland et al. 2020). One issue within the prediction view is about the format of these predictions. Without specifying whether the predictions involve concepts, parts of speech, or expected grammatical structures, specific lexical entries, or phonological or orthographic forms, the proposal remains rather generic and difficult to falsify. For this reason, empirically demonstrating the anticipated activation of specific representations has significant theoretical implications.

One of the most compelling pieces of direct empirical evidence of phonological prediction was reported by DeLong et al. (2005). Their experiment leveraged the English phonological rule in which the indefinite article appears as “*a”* before consonant‐initial words and “*an”* before vowel‐initial words. Participants read sentences with varying levels of contextual constraint that led to expectations of either a consonant‐ or vowel‐initial word. The authors examined the modulation of the N400 amplitude elicited by the article, which could either match or mismatch the phonological form of the predicted noun. Results showed that N400 amplitude decreases as a function of increasing *cloze probability*, both for the target noun and, critically, for the preceding article. This suggests that participants predicted the phonological form of the upcoming word, leading to increased negativity when the article mismatched the expected form. Despite the theoretical significance of the DeLong et al. (2005) findings, subsequent attempts to replicate the N400 modulation on the pre‐target article have yielded mixed results (Ito et al. 2017, 2020; Martin et al. 2013; Nicenboim et al. 2020; Nieuwland et al. 2018).

Laszlo and Federmeier (2009) investigated the prediction of phonological/orthographic word forms using stimuli phonologically/orthographically related to the predictable word. They found that the N400 amplitude was larger for unexpected words and non‐words compared to predictable words. Crucially, the N400 effect was reduced for form‐related words and non‐words relative to form‐unrelated words and non‐words. Similarly, Ito et al. (2016) used a paradigm in which participants read sentences ending with either a predictable word (e.g., book), a form‐related word (e.g., hook), a semantically related word (e.g., page), or an unrelated word (e.g., sofa). They replicated the reduced N400 effect for form‐related words, but this effect was found only for high‐cloze sentence continuations and slow presentation rates (700 ms per word). The authors interpreted their results as supporting *prediction‐by‐production* accounts of linguistic prediction. Prediction‐by‐production models propose that prediction during comprehension relies on the same representations and mechanisms as those used in language production (Federmeier 2007; Gastaldon et al. 2024; Huettig 2015; Pickering and Gambi 2018; Pickering and Garrod 2007, 2013). According to this view, comprehenders covertly imitate what they hear, generating production‐based representations of upcoming speech (Pickering and Garrod 2013). This prediction mechanism involves a hierarchical pre‐activation of linguistic representations, from semantic to phonological forms, as in language production (Levelt 1999). Notably, phonological word‐form pre‐activation occurs only when sufficient time and cognitive resources are made available by the specific task and paradigm. DeLong et al. (2019, 2021) employed the same experimental design as Ito et al. (2016), but with different materials, and reported a reduced N400 effect for form‐related words at both 500 and 700 ms per word presentation rates. They interpreted their findings as suggesting that phonological prediction does not necessarily require production‐like processing, which is constrained by timing and resources. Instead, pre‐activation of upcoming words could arise from passive *spreading of activation* between linguistic representations (Anderson 1983; Collins and Loftus 1975; Huettig et al. 2022; Hutchison 2003; McRae et al. 1997). Within this framework, linguistic representations can partially activate networks of related items (semantically, associatively, or phonologically; Pickering and Gambi 2018). This mechanism is thought to be less constrained by time and/or processing resources and largely independent of the specific linguistic context, although some accounts suggest it may still be sensitive to contextual details (Huettig et al. 2022). Importantly, the proposal that spreading of activation can lead to phonological form pre‐activation is not incompatible with prediction‐by‐production theories. Pickering and Gambi (2018) proposed that linguistic pre‐activation could rely on both *prediction‐by‐production*, which is cognitively demanding and optional, and *prediction‐by‐association*, which is automatic but less precise. The findings of DeLong et al. (2019, 2021) suggest that phonological form pre‐activation can occur through mechanisms outside the language production network, or that phonological form pre‐activation within a prediction‐by‐production model may occur more rapidly than previously thought.

Further studies have reported ERP (and ERF, event‐related fields, for MEG) predictability effects preceding the N400, which have been interpreted as evidence of the pre‐activation of sublexical representations. However, their replicability remains weak and inconsistent (for a review, see Nieuwland 2019).

Finally, phonological prediction has also been explored using behavioral techniques, such as the *visual world paradigm* in eye‐tracking studies. These studies examined whether a phonological competitor of a predictable target word attracts more anticipatory looks than phonologically or semantically unrelated distractors. Results from this paradigm have been inconsistent across studies (Ito 2019, 2018; Ito and Husband 2017; Ito and Sakai 2021; Kukona 2020; Li et al. 2022; Li and Qu 2024; Zhao et al. 2024). Nonetheless, a recent meta‐analysis (Ito 2024), reported a small but reliable phonological competitor effect.

In a previous behavioral study (Sala et al. 2024), we investigated whether speaker identity (L1‐ vs. L2‐accented^1^) influences phonological predictions. Participants were first familiarized with an L1‐ and an L2‐accented speaker. Then they silently read written sentence frames that were either highly or weakly constraining toward an upcoming spoken target word, which was pronounced either by the L1‐ or the L2‐accented speaker. The L2‐accented speaker made consistent phonological errors on the first phoneme of the target word. Crucially, during the trial presentation, speaker identity was either cued or not with an image of the speaker's face. Participants performed a lexical decision task on the spoken target word, and in the L2‐accented condition, they were explicitly instructed to accept mispronounced words as correct. Our results showed that cueing the speaker identity led to faster RTs (response times) for predictable words but not for unpredictable ones, suggesting that participants used the face cue to implement phonological predictions.

The present study aims to investigate this face‐cueing effect using ERPs. We adapted the experimental procedure of the previous behavioral study to the ERP context. We hypothesized that the face cue would facilitate phonological predictions, aiding phonological encoding of the target word and consequently the retrieval of the corresponding lexical‐semantic representation. Therefore, cueing speaker identity should be associated with a greater N400 predictability reduction compared to when the speaker identity is not cued, reflecting easier processing of predictable words due to phonological pre‐activation. Additionally, we aim to explore whether the use of speaker's accent information in prediction differs between the L1‐ and the L2‐accented conditions. In the L1‐accented condition, participants should anticipate a standard phonology, whereas in the L2‐accented condition, they should be able to anticipate unusual specific phonological deviations.

## 2 Methods

### 2.1 Participants

A total number of 48 healthy participants (39 women) took part in the study, aged between 18 and 30 years with a mean age of 23.27 ± 3.05 years and with a mean education level of 16.33 ± 1.94 years. Participants were recruited from healthy volunteers and students at the University of Padova and received 15 euros for their participation. Participants were right‐handed L1 Italian speakers with no history of neurological, language‐related, or psychiatric disorders. Our initial sample size of 48 participants aimed to ensure sufficient power to detect at least the effect of face cues on predictable words after possible data loss. The power analysis, conducted using the method proposed by Judd et al. (2016), indicated that at least 38.9 participants would be required to detect a small‐to‐medium effect (Cohen's *d* = 0.3) with 80% power. The research adhered to the principles outlined in the Declaration of Helsinki. Participants provided their informed consent before participating in the experiment. The research protocol was approved by the Ethics Committee for Psychological Research of the University of Padova (protocol number: 5181).

### 2.2 Materials

The materials are available via the Open Science Framework (OSF) repository of the current project (https://osf.io/brvkx/). The target stimuli consisted of 168 spoken words (mean length = 5.86 ± 1.86 phonemes) beginning with the phonemes /r/, /p/, and /k/. These three phonemes were not present in any other position within words. Each target word was preceded by a written sentential context that could be either high constraining (HC) or low constraining (LC) toward the target word. Below are two illustrative examples for the same target word (*conigli*/*rabbits*). In (1a), the context strongly constrains the possible sentence continuations, making the target word highly predictable. In contrast, the context in (1b) allows for a broader range of continuations, making the target word less predictable.

(1a) La carota è il cibo preferito dei conigli (*The carrot is the favorite food of rabbits*).

(1b) Al parco abbiamo visto una famiglia di conigli (*At the park we saw a family of rabbits*).

To determine the constraint level, an online sentence completion questionnaire was administered to 22 participants, who did not take part in the experiment. They were instructed to complete each sentence frame with the first word that came to their mind. The sentence constraint was operationalized as the proportion of total responses involving the most frequent continuation (*High Constraint*: mean_sentence constraint_ = 0.94 ± 0.07; *Low Constraint*: mean_sentence constraint_ = 0.17 ± 0.09). The target word in HC sentence frames was always the most frequent continuation (*High Constraint*: mean_cloze probability_ = 0.94 ± 0.07), while in LC sentence frames it was a semantically plausible continuation (*Low Constraint*: mean_cloze probability_ = 0.03 ± 0.09). The sentence frames varied in length (mean = 9.37 ± 2.11 words, range = 4–15 words), but their length was matched between conditions (HC sentence frames: mean = 9.45 ± 2.20 words, range = 4–15 words; LC sentence frames: mean = 9.28 ± 2.03 words, range = 4–15 words; *p =* 0.455).

The target stimuli were spoken by an artificial voice with an L1 Italian accent in one condition and an artificial voice with an artificially built L2 accent in the other condition. The artificial L2 accent was created by manipulating either the place or manner of articulation of the target phonemes, as in Sala et al. (2024). Specifically, three frequent word‐initial phonemes (/r/, /p/, and /k/) were produced by the L2‐accented voice as /l/, /b/, and /ɢ/, respectively. This manipulation aimed to introduce systematic phonological variability between the two speakers and to control participants' familiarity with different L2 accents. The speech stimuli were synthesized using the Microsoft Azure text‐to‐speech service. The prebuilt neural voice of an Italian speaker (Fabiola) was selected for the L1‐accented voice. The prebuilt neural voice of an Indian speaker (Neerja) was selected for the L2‐accented voice. The two speakers were selected with the aim of making participants associate a given face stimulus with an L1/L2 speaker voice. The stimuli were synthesized starting from the IPA transcription of the words in Italian in order to minimize the impact of the phonetic variability between the L1 and the L2 voice. The phonological manipulation of the L2‐accented speaker was implemented by changing the target phonemes in the IPA transcription of the stimuli (e.g., the target stimulus was /kˈaldo/ for the L1 speaker voice and /g'aldo/ for the L2 speaker voice). In this way, both the L1 and the L2 speakers received the same phonetic sequences as input, except for the phonological manipulation. The L2‐accented voice mispronounced the initial phoneme of all target stimuli. The mispronounced words did not correspond to the pronunciation of any existing Italian word. The phonological manipulation was implemented to synthesize both the experimental stimuli and the familiarization speech of the L2 speaker. The spoken target stimuli had a similar duration between L1 and L2 accent conditions (L1 accent: 688 ± 129 ms; L2 accent: mean = 700 ± 125 ms; *p* = 0.399). A more detailed description of the artificial L2 accent and of the stimuli generation procedure is reported in Sala et al. (2024).

Two face stimuli were selected from the AMFD (American Multiracial Face Database; Chen et al. 2021) to represent the L1‐accented speaker and the L2‐accented speaker. The AMFD includes face stimuli that have been rated by naïve observers on several dimensions, including “racial group” membership, evaluated on a 7‐item Likert scale ranging from 1 (very atypical) to 7 (very typical). For the L1‐accented speaker, we selected a face stimulus that we considered Italian‐looking (White: mean = 6.22 ± 0.82) and evaluated as not typical in the other norms (Asian: mean = 1.65 ± 1.11; Black: mean = 1.68 ± 1.33; Latino/a: mean = 3.30 ± 1.73; Middle Eastern: mean = 2.57 ± 1.77; Biracial/Multiracial: mean = 3.62 ± 1.83). For the L2‐accented speaker, we selected a face stimulus evaluated as more typical for norms different from White/Caucasian (White: mean = 2.81 ± 1.73; Asian: mean = 2.54 ± 1.56; Black: mean = 2.56 ± 1.65; Latino/a: mean = 5.28 ± 1.19; Middle Eastern: mean = 4.40 ± 1.75; Biracial/Multiracial: mean = 4.60 ± 1.44). The two‐face stimuli had similar ratings in the other dimensions evaluated (see Table 1).

**TABLE 1:** AMFD ratings for the L1‐ and L2‐accented speaker face stimuli.

	L1 speaker	L2 speaker
Attractive	4.97 ± 1.09	4.53 ± 1.09
Dominant	2.97 ± 1.85	2.79 ± 1.48
Emotional expression	5.78 ± 0.82	5.82 ± 0.93
Racial ambiguity	2.86 ± 1.62	3.89 ± 1.37
Smart	5.24 ± 1.09	5.09 ± 1.11
Smile	5.11 ± 1.66	5.23 ± 1.63
Trust	5.00 ± 1.13	5.21 ± 1.32
Warm	5.19 ± 1.17	5.58 ± 1.08

### 2.3 Procedure and Design

**FIGURE 1:**
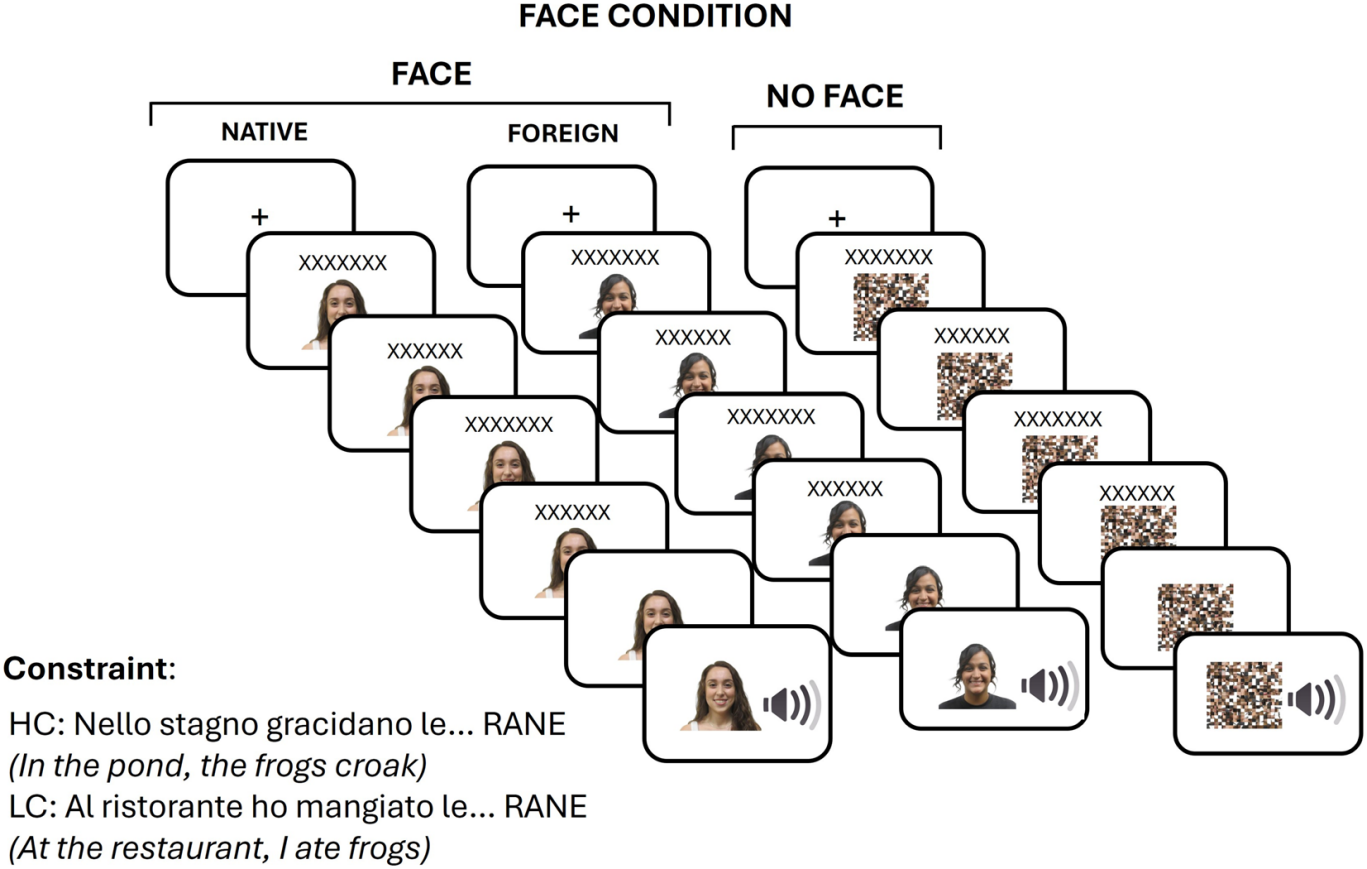
Schematic representation of the experimental paradigm and procedure. A trial consisted of a variable number of frames presented for 500 ms each (300 ms word duration, 200 ms inter‐word interval). The number of frames depended on the length of each sentence. In each frame, a word was presented together with a visual stimulus that could be either the face of the L1 or L2 speaker or a control stimulus. The accent of the target word (in the example RANE/frogs) presented auditorily could be cued or not by an image of the speaker's face. Sentences could be highly constraining (HC) or low constraining (LC) toward the target word.

The experiment was carried out using PsychoPy (Peirce et al. 2019). Participants were seated in a comfortable chair in a soundproof room with a computer connected to an LCD monitor, speakers, and a keyboard. During the familiarization phase, participants viewed a picture of the speaker's face and listened to a one‐minute speech in which the speaker introduced herself (see Sala et al. 2024). All participants were exposed to both speakers' faces and listened to the corresponding accented speech. In the experimental phase, participants were instructed to silently read the sentence frames displayed on the screen. The speaker's face was presented 2000 ms before sentence onset, 4.5 cm below the center of the screen, and remained continuously visible throughout the whole trial. In half of the trials, the face was replaced by a single control stimulus, created by scrambling together the faces of the two speakers (see Figure 1). This approach ensured that the control stimulus was identical across both accent conditions and could not provide any information allowing participants to predict the speaker's accent. Both the face and the control stimulus were 10 cm wide and 10 cm high. The presentation of each sentence frame started with a fixation cross appearing 4.5 cm above the center of the screen for 300 ms (450 ms before sentence onset). The sentence frames were presented word‐by‐word at a regular pace (300 ms word duration, 200 ms inter‐word interval). We planned to present the target word 800 ms after the presentation of the last word of the sentence; however, due to a technical error, this interval resulted in being 930 ms. A fixation cross followed the last word of the sentence frame. Participants were instructed to avoid blinks and eye movements during the experimental trials. Each trial was followed by a 1750 ms interval in which participants could blink.

In 25% of the trials, a written question was presented to participants, asking them to judge whether they expected the word produced by the speaker or not, regardless of how it was pronounced. Participants were instructed to categorize the spoken targets as expected or not expected by pressing the ‘M’ or ‘C’ keys on the keyboard with their left and right index fingers. In half of the questions, they were asked to respond to expected words with the index finger of their dominant hand. In the other half, they were asked to respond to unexpected words with the index finger of their dominant hand.

Before starting the experimental session, participants completed 8 practice trials that were not part of the experimental materials. The whole experimental session lasted about 2 h. Each participant was presented with 336 trials, and they had the possibility to take a break every 21 trials. Each target word was presented twice, once in a high‐constraining sentence frame and once in a low‐constraining sentence frame. To avoid close repetitions, the experimental list was divided into two blocks, with each target word spoken in a different accent across blocks and appearing only once per block. The speaker's accent was either cued or not cued by the speaker's face, resulting in 42 trials per experimental condition. We created four experimental lists in order to present each target word (predictable or unpredictable) in the different accent and face conditions according to a Latin square design. Twelve participants were assigned to each list. The order of the two blocks was counterbalanced between participants, and the order of the trials within blocks was randomized.

### 2.4 EEG Data Acquisition and Preprocessing

**FIGURE 2:**
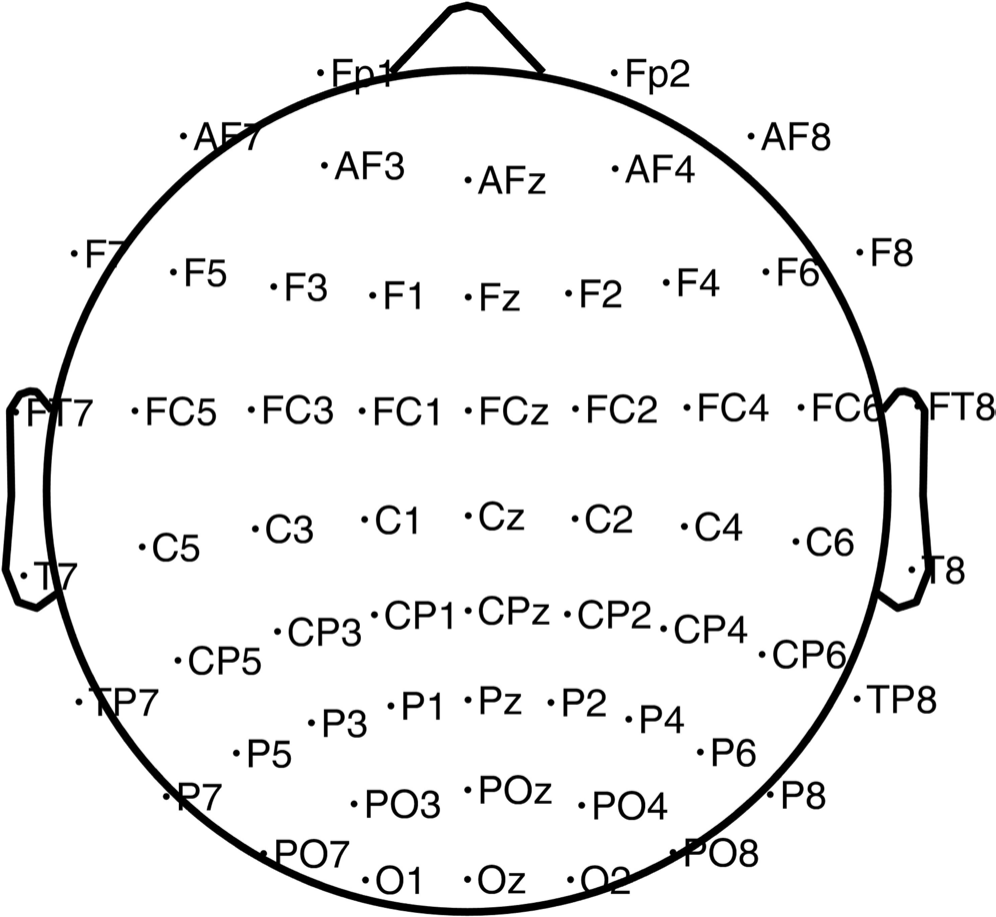
2D electrode layout used for the current experiment. The setup also included three electrodes used to record blinks and saccades, and an electrode positioned on the right earlobe.

Electroencephalogram was recorded with a system of 64 active Ag/AgCl electrodes (Brain Products), placed according to the 10–20 convention (ActiCap). Sixty of them were used as active electrodes (Fp1, Fp2, AF3, AF4, AF7, AF8, AFz, F1, F2, F3, F4, F5, F6, F7, F8, Fz, FT7, FT8, FC1, FC2, FC3, FC4, FC5, FC6, FCz, T7, T8, C1, C2, C3, C4, C5, C6, Cz, TP7, TP8, CP1, CP2, CP3, CP4, CP5, CP6, CPz, P1, P2, P3, P4, P5, P6, P7, P8, Pz, PO3, PO4, PO7, PO8, POz, O1, O2, Oz). The reference electrode was placed on the left earlobe. Three electrodes were used to record blinks and saccades (external ocular canthi and below the left eye), and one was positioned on the right earlobe. Figure 2 shows the scalp position of the electrodes.

The setup was deemed acceptable if electrode impedance was below 20 kΩ at the end of electrode placement. The signal was amplified and digitized at a sampling rate of 500 Hz. Before the tasks, a resting state of 3 min was recorded. Descriptive statistics for the participants' performance in the behavioral task were computed to ensure that participants were indeed reading sentences and listening to words. One participant classified a high number of words (69%) in low‐constraint contexts as “predictable” and therefore was excluded.

Preprocessing was performed in MATLAB using EEGLAB (Delorme and Makeig 2004) and Fieldtrip (Oostenveld et al. 2011). EEG signals were offline re‐referenced to the average activity of the right and left earlobes. A band‐pass filter (0.5–80 Hz) was applied to the raw data. Noisy or flat channels were excluded (max 3 channels per participant). Segments with extreme muscle artifacts were also excluded. Subsequently, independent component analysis (ICA) with dimensionality reduction to 60 components (PCA) was computed to detect and remove artifacts with known time series and topographies (blink, saccades, and power‐line noise at 50 Hz). ICA was applied to band‐pass filtered data (1–55 Hz) to optimize the identification of ocular artifacts and power‐line noise. ICA weights were applied to the 0.5–80 Hz filtered data. Missing channels were interpolated (superfast spherical interpolation) after ICA correction. After a low‐pass filter (30 Hz), 1200‐ms epochs were extracted starting from 200 ms before the onset of the auditory stimuli. A pre‐stimulus 200 ms baseline correction was applied to the extracted epochs. Trials including slow drifts, muscular activity, or remaining ocular artifacts were manually removed based on visual inspection (see Table 2). Two participants were excluded from the analyses due to the high number of rejected epochs (> 20%). Three more participants were excluded due to high residual alpha activity. The final sample included 42 participants (mean _age_ = 23.05 ± 2.97 years, 34 women).

**TABLE 2:** Accepted trials (mean and SD) for the different experimental conditions.

	High constraint		Low constraint	
	Face	No face	Face	No face
L1 accent	41.02 ± 1.29	41.02 ± 1.26	41.19 ± 1.09	40.98 ± 1.32
L2 accent	41.07 ± 1.47	40.83 ± 1.36	41.12 ± 1.40	41.02 ± 1.57

### 2.5 Statistical Analyses

The statistical analyses were performed using the open‐source software R (R Core Team 2023). Analyses focused on an a priori determined N400 time window (300–500 ms after word onset) in a cluster of centro‐parietal electrodes (Cz, C3, C4, Pz, P3, P4), where predictability effects have been consistently reported (Kutas and Federmeier 2011; Nieuwland et al. 2018; Šoškić et al. 2022). The single‐trial mean voltage in the N400 time window was analyzed using linear mixed‐effect models (Bates et al. 2015). We used a hierarchical model comparison approach to identify the best‐fitting model for our data (Heinze et al. 2018). The model comparison was based on the Akaike Information Criterion (AIC) and especially delta AIC and AIC weight as indexes of the goodness of fit. The AIC and AIC weight give information on the models' relative evidence (i.e., likelihood and parsimony); therefore, the model with the lowest AIC and the highest AIC weight is to be preferred (Wagenmakers and Farrell 2004). The model comparison included a baseline model with Constraint by Participant random slopes and Participant and Item as random intercepts to account for participant‐specific variability and item‐specific variations (Baayen et al. 2008). The predictors' order was established, giving priority to the main effects over interactions and to the effects (i.e., Accent and Constraint) previously reported in the literature (Goslin et al. 2012; Grey and van Hell 2017; Kutas and Federmeier 2011). The inclusion of predictors followed this order: (i) Accent (L1 vs. L2); (ii) Constraint (HC vs. LC); (iii) Face (Face vs. No Face); (iv) Accent*Constraint; (v) Constraint*Face; (vi) Accent*Face; (vii) Accent*Constraint*Face. Main effects were estimated using sum coding (Brehm and Alday 2022). The critical alpha was set to 0.05. Post hoc comparisons were performed using the contrast function of the emmeans package (Lenth et al. 2023). The *p*‐values were adjusted using Bonferroni's correction (Bonferroni 1936).

## 3 Results

### 3.1 Behavioral Results

In 25% of the trials, a written question was presented to participants, asking them to judge whether they expected the word produced by the speaker or not, regardless of how it was pronounced. The proportion of target words classified as “predictable” in control trials for the different experimental conditions is reported in Table 3.

**TABLE 3:** Proportion of target words classified as “predictable” in the different experimental conditions.

Accent	High constraint		Low constraint	
	Face	No face	Face	No face
L1	0.94 ± 0.23	0.93 ± 0.25	0.07 ± 0.26	0.08 ± 0.27
L2	0.95 ± 0.23	0.94 ± 0.24	0.05 ± 0.23	0.07 ± 0.25

Participants seem to categorize most of the words in control trials with high constraint contexts as *predictable* and most of the words in control trials with low constraint contexts as *not predictable*, suggesting that they were paying attention to the stimuli. Moreover, this judgment doesn't seem to be influenced by the presence of the face cue (see Supporting Information for statistical analyses).

### 3.2 ERP Results

#### 3.2.1 Visual Inspection of Waveforms

**FIGURE 3:**
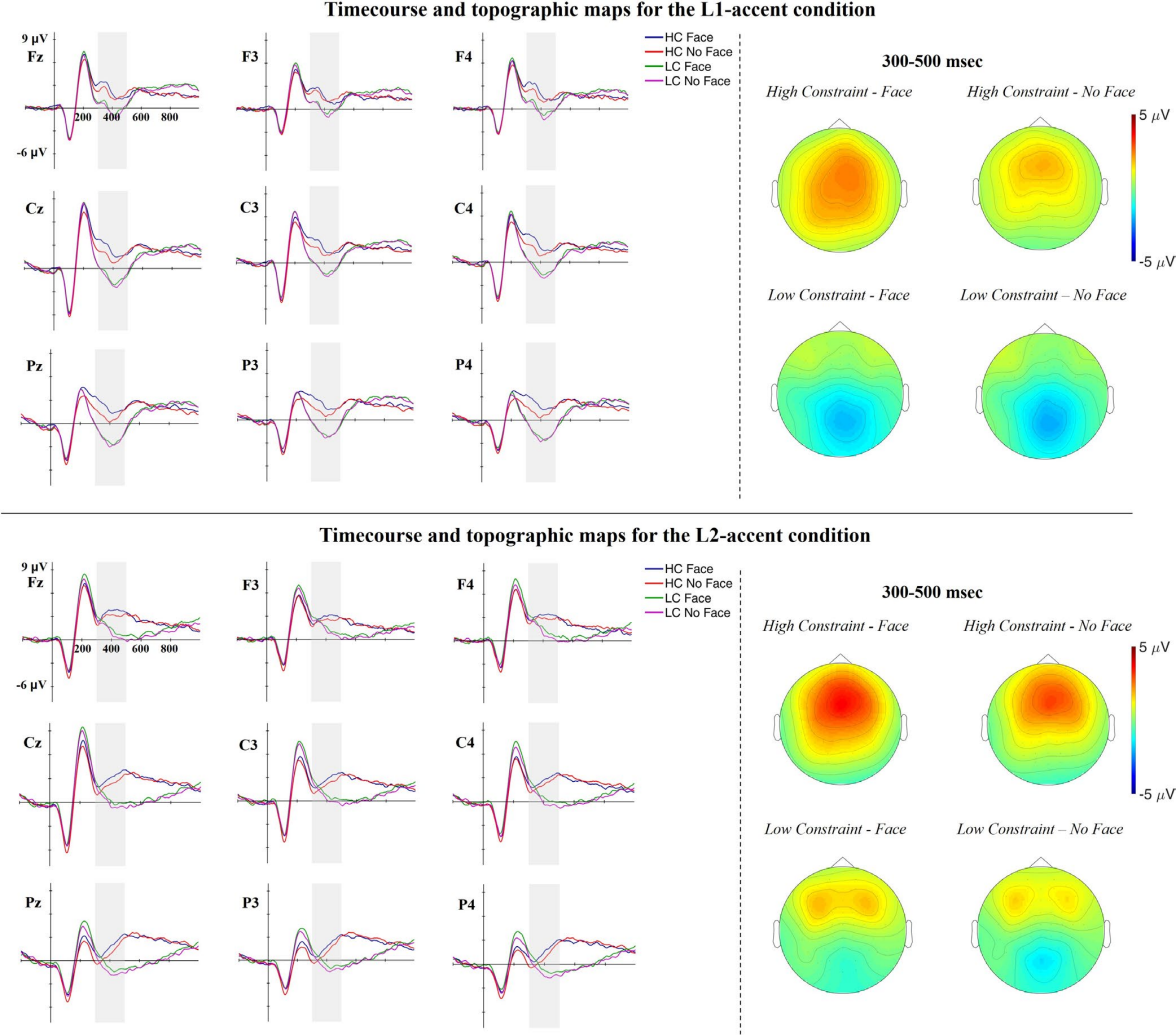
ERPs time course and topographic maps for the L1‐ and the L2‐accented conditions. The gray pattern marks the N400 time window (300–500 ms). The 9 electrodes were chosen to represent the EEG signal in frontal, central, and parietal regions and across the left, midline, and right hemispheres based on our electrode layout.

Figure 3 shows the grand‐average ERP waveforms in 9 representative electrodes (Fz, F3, F4, Cz, C3, C4, Pz, P3, P4) and the topographic maps of the experimental conditions.

**FIGURE 4:**
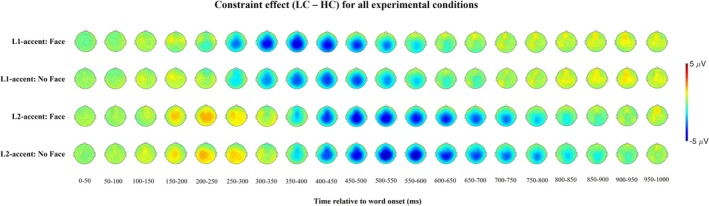
Topographic maps of the constraint effect according to accent and face conditions.

As predicted, we observed differences in the N400 time window according to the constraint condition that are modulated by the presence of the face. However, the constraint effect is present also in previous and successive time intervals, and it seems to be different across the L1 versus L2 accented condition (Figure 4).

Compared to predictable words, unpredictable words in the L1‐accent condition present larger centro‐parietal negativity starting from 250 to 300 ms. In the L2‐accent condition, unpredictable words present larger positivity between 150 and 300 ms and larger centro‐parietal negativity from 350 to 400 ms. Given this complex pattern, we performed two types of analyses. A first a priori planned analysis on the N400 time window and a second post hoc Temporal Exploratory Factor Analysis (EFA) to better characterize the ERP components underlying the effects observed after word onset. We computed separate EFAs for the L1‐ and the L2‐accented conditions, as visual inspection of the grand‐average waveforms points to noticeable differences in the ERP components elicited by these conditions.

#### 3.2.2 N400: 300–500 ms

As reported in Table 4, the best‐fitting model for N400 amplitude is Model 5:

$$\begin{array}{c} \text{Amplitude}\sim \text{Accent}+\text{Constraint}+\text{Face}+{\text{Constraint}}^{*}\text{Accent}\\ +{\text{Constraint}}^{*}\text{Face}+\left(\text{Constraint}|\text{Participant}\right)+\left(1|\text{Item}\right) \end{array}$$

**TABLE 4:** The comparison of LMER models predicting the N400 amplitude.

Models	Deviance	dAIC	AICw
M0. Amplitude ~ (Constraint|Participant) + (1|Item)	89,293.77	166.67	0.0
M1. Amplitude ~ Accent + (Constraint|Participant) + (1|Item)	89,239.48	114.38	0.0
M2. Amplitude ~ Accent + Constraint + (Constraint|Participant) + (1|Item)	89,179.38	56.29	0.0
M3. Amplitude ~ Accent + Constraint + Face + (Constraint|Participant) + (1|Item)	89,150.66	29.56	0.0
M4. Amplitude ~ Accent + Constraint + Face + Constraint*Accent + (Constraint|Participant) + (1|Item)	89,121.36	2.26	0.15
M5. Amplitude ~ Accent + Constraint + Face + Constraint*Accent + Constraint*Face + (Constraint|Participant) + (1|Item)	**89,117.09**	**0.0**	**0.46**
M6. Amplitude ~ Accent + Constraint + Face + Constraint*Accent + Constraint*Face + Accent*Face + (Constraint|Participant) + (1|Item)	89,116.87	1.78	0.19
M7. Amplitude ~ Accent + Constraint + Face + Constraint*Accent + Constraint*Face + Accent*Face + Accent*Constraint*Face + (Constraint|Participant) + (1|Item)	89,114.73	1.63	0.20

Abbreviations: AICw, AIC weight; dAIC, difference between AIC of each model and the model with lower AIC; deviance, residual deviance. Significance use of bold is used to highlight the best fitting model.

**FIGURE 5:**
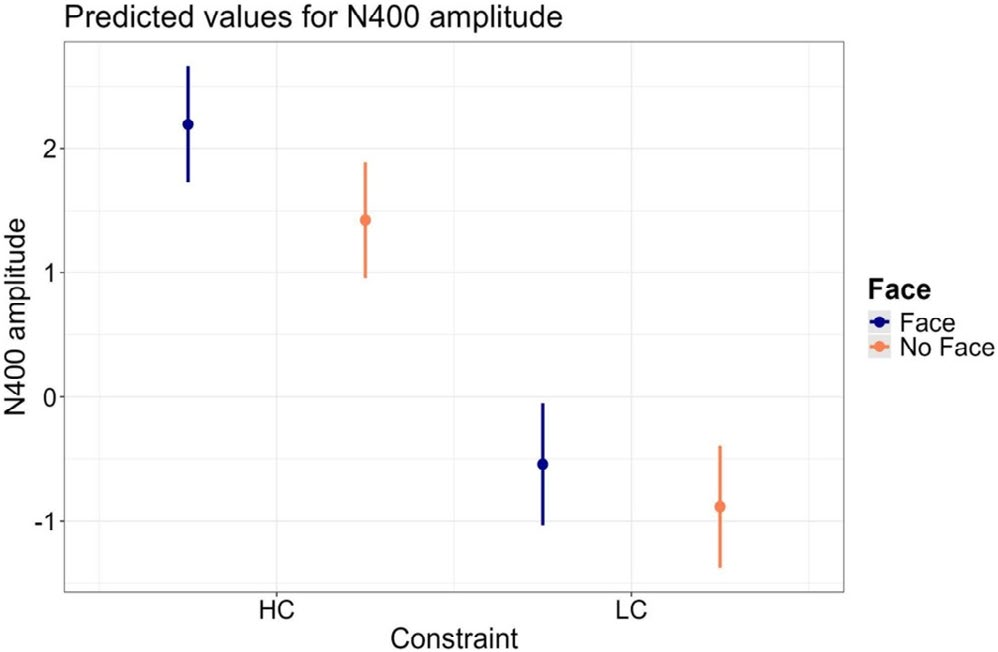
Model estimates for the interaction between Constraint*Face. The error bars indicate 95% confidence intervals.

The results of the best‐fitting model (Model 5) are reported in Table 5. All predictors showing main effects are implied in interactions; therefore, we focus on the latter. The interaction between Constraint and Accent indicates that the Constraint effect, namely smaller N400 amplitude for predictable words compared to unpredictable words, is larger for the L1‐ compared to the L2‐accented condition. Most importantly, the interaction between Constraint*Face indicates that cueing the speaker identity is associated with an increased Constraint effect (Figure 5). Post hoc comparisons showed that cueing the speaker identity is associated with smaller N400 amplitude for predictable (*b* = 0.770, *z*‐ratio = 5.258, *p* < 0.001) but not for unpredictable words (*b* = 0.343, *z*‐ratio = 2.343, *p* = 0.076).

**TABLE 5:** Model results for the best‐fitting model for the N400 amplitude.

	Estimate	CI (95%)	Std. error	*t*‐value	*p*
Intercept	0.548	[0.143 0.952]	0.204	2.683	0.010
Accent[L2]	0.381	[0.280 0.483]	0.052	7.383	< 0.001
Constraint[HC]	1.260	[1.036 1.484]	0.113	11.135	< 0.001
Face[Face]	0.278	[0.177 0.380]	0.052	5.376	< 0.001
Accent[L2]*Constraint[HC]	−0.280	[−0.381 –0.179]	0.052	−5.417	< 0.001
Constraint[HC]*Face[Face]	0.107	[0.005 0.208]	0.052	2.064	0.039

### 3.3 Interim Discussion

In the present study, we aimed to investigate whether sentence contexts could be used to predict the phonological form of a highly predictable word. To address this, we capitalized on the fact that L2‐accented speakers often exhibit phonological errors to examine whether the prediction system can account for the phonological variability across speakers. In our experimental paradigm, participants read sentence frames in which the last word was produced by an L1‐ or an L2‐accented speaker. The last word of the sentence could be predictable or not based on sentence context. Most importantly, during trial presentation, speaker identity could be cued or not by an image of the speaker's face, thus manipulating the availability of information about the phonological features of the upcoming target word before its presentation. We hypothesized that cueing speaker identity should be associated with a greater N400 predictability effect, reflecting easier processing of predictable words due to the pre‐activation of the phonological word form.

The findings from the main analysis supported our hypothesis: unpredictable words elicited a larger N400 compared to predictable words, and this N400 effect was larger when speaker identity was cued compared to when it was not. Crucially, post hoc analyses showed that the face cue reduced the N400 amplitude for predictable words but not for unpredictable words. We found no evidence that the observed face‐cueing effect was modulated by the accent of the speaker (L1 vs. L2). This result nicely aligns with previous behavioral data (Sala et al. 2024), further corroborating the involvement of phonological representations in linguistic prediction and suggesting that predictive processes are sensitive to interindividual differences in phonological features.

### 3.4 Temporal Exploratory Factor Analysis

**FIGURE 6:**
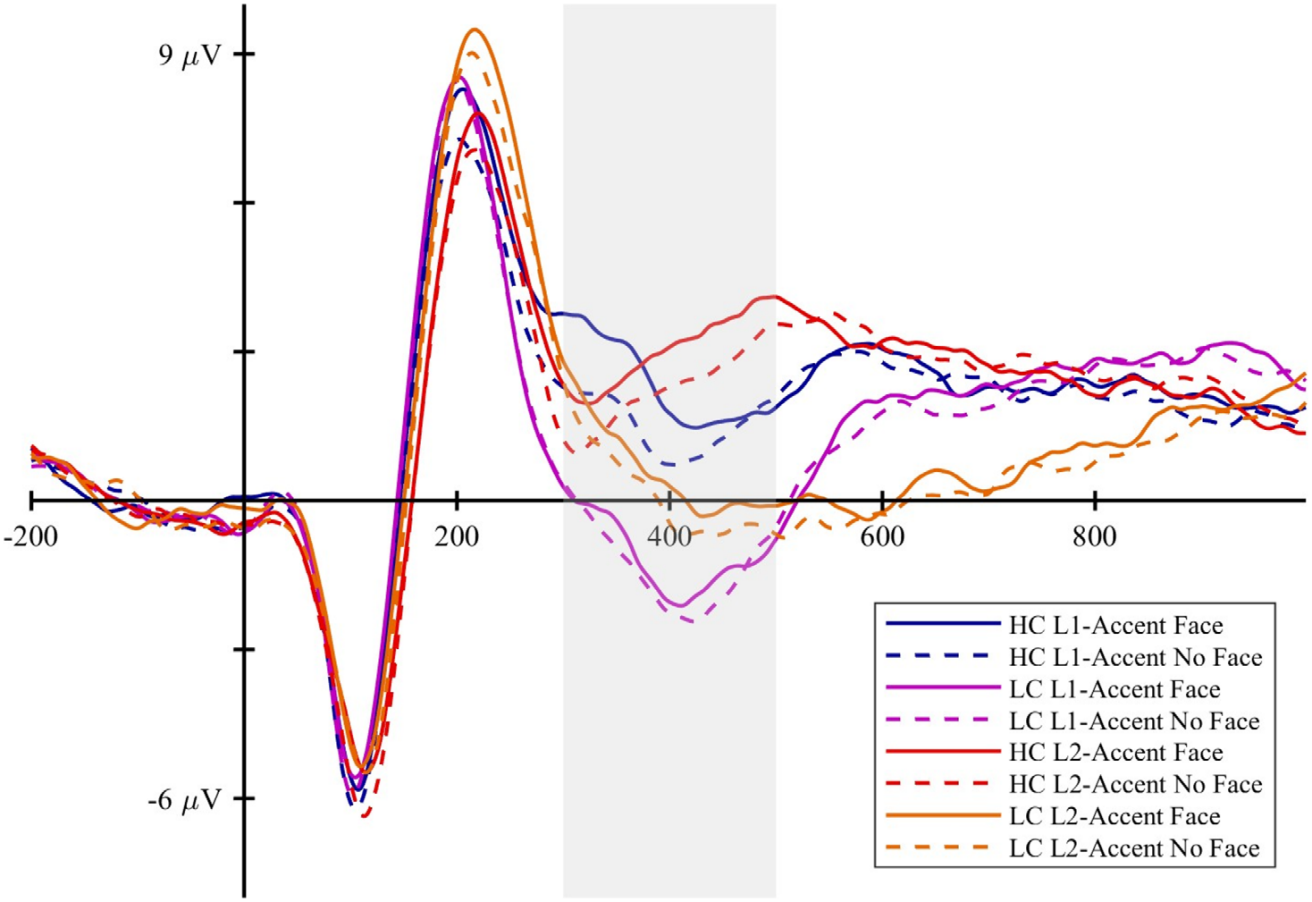
ERPs timecourse in Cz electrode for all the experimental conditions, with the N400 time window (300–500 ms) highlighted.

Figure 6 illustrates the grand‐average ERP in Cz electrode for all the experimental conditions. Visual inspection of the waveforms reveals a more complex pattern than hypothesized. First of all, starting from 300 ms after target onset, the non‐subtracted waveforms seem to present a different shape for L2‐accented words compared to L1‐accented words. Moreover, the face‐cueing effect for predictable words seems to emerge prior to the N400 time window, with positive deflections within the analyzed time window.

To better characterize the ERP components observed in our study, we conducted a Temporal Exploratory Factor Analysis (EFA). This approach was chosen because the traditional method of analyzing the observed signal average across fixed time windows has limitations, as it assumes clear spatial and temporal separation between ERP components, which is not always the case (Luck 2014). Temporal EFA is a statistical decomposition technique that can be used to identify and disentangle the constituent components of the observed ERPs (Dien 2012; Dien and Frishkoff 2005; Scharf et al. 2022).^2^ Temporal EFA allows the extraction of *factors* representing estimates of the underlying ERP *components*, based on statistical associations between sampling points. Factors are described by two coefficients: *factor loadings*, which represent the factor's contribution to the voltage at a specific sampling point, and *factor scores*, which quantify the factor's contribution for a specific observation (for more details, see Scharf et al. 2022).

Temporal EFA was computed following the procedure reported by Scharf et al. (2022). Average waveforms for each participant, electrode, and condition were obtained by averaging the single trials. The data were resampled at 250 Hz before running the Temporal EFA. Separate EFAs were computed for the L1‐ and L2‐accented conditions, as visual inspection of the grand‐average waveforms points to noticeable differences in the ERP components elicited by these conditions. Conducting separate EFAs is preferable when *measurement invariance* cannot be assumed (Beauducel and Hilger 2018; Meredith 1993; Möcks 1986), such as in cases of condition‐related component structures or latency differences (Barry et al. 2016). Temporal EFA was computed on the trials‐averaged waveforms, including the baseline pre‐stimulus period and all the EEG channels (Dien 2012). The EFA model was estimated using the covariance matrix of the sample points. The number of factors to be extracted was based on the Empirical Kaiser Criterion (EKC) (Braeken and van Assen 2017; Li et al. 2020). The rotated solution was estimated using Geomin rotation (Yates 1987) with 30 random start values and the rotation parameter ϵ set to 0.01. We considered for the analysis only factors explaining more than 3% of the total variance.

We analyzed the *peak amplitude* (factor scores multiplied with the peak factor loading) of the factors reflecting ERP components of interest. We selected a cluster of centro‐parietal electrodes (Cz, C3, C4, Pz, P3, P4) for factors reflecting components with a centro‐parietal distribution (P3b and N400), while a cluster of fronto‐central electrodes (Cz, C3, C4, Fz, F3, F4) was selected for factors reflecting components with a fronto‐central distribution (N1, P2, P3a). We also explored factors reflecting slow waves extending beyond the N400 time window. Given the heterogeneity of the scalp distribution of slow waves (Van Petten and Luka 2012), we selected a centro‐parietal or a fronto‐central electrode cluster based on the anterior or posterior distribution of the factor of interest. For each factor, we run a repeated measures ANOVA, testing for the following effects: (i) Constraint, (ii) Face, (iii) Constraint*Face. Post hoc comparisons were performed using the contrast function of the emmeans package (Lenth et al. 2023), with *p*‐values adjusted using Bonferroni correction (Bonferroni 1936).

#### 3.4.1 L1‐Accent Temporal EFA

In the L1‐accent condition, we extracted a 23‐factor solution explaining 95% of the total variance. The unstandardized factor loadings for all extracted factors are reported in the Supporting Information.

**FIGURE 7:**
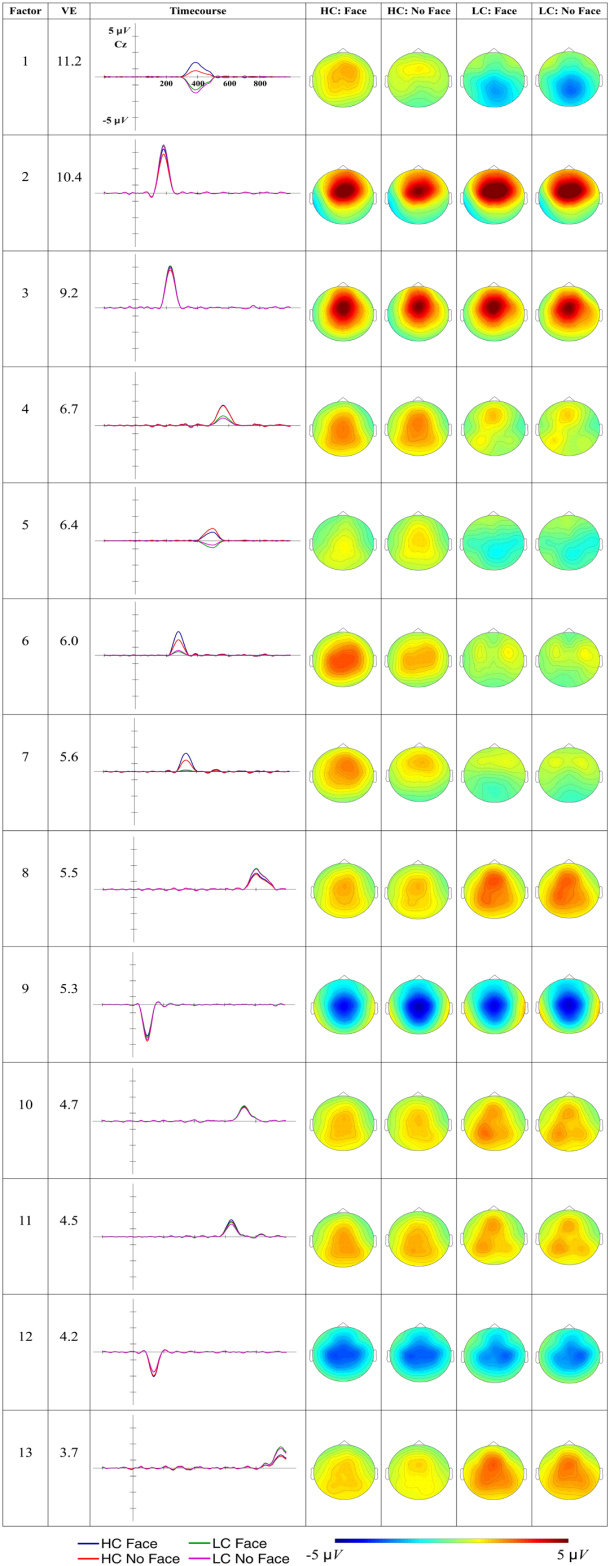
Time course and scalp distribution of the ERPs reconstructed factors from the L1‐accent Temporal EFA in the different experimental conditions. VE: percentage of total variance explained by the extracted factor. The reconstructed ERP time course at the electrode Cz and the scalp distribution of the peak amplitude (factor scores multiplied with the peak factor loading) are reported.

The factors explaining more than 3% of the total variance are shown in Figure 7.

**FIGURE 8:**
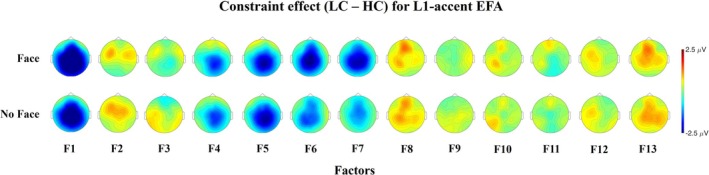
Topographic maps of the Constraint effect for factors explaining more than 3% of variance in the L1‐accent Temporal EFA.

Figure 8 presents the difference topographic maps for the Constraint effect in the factors explaining more than 3% of variance.

Factors from 1 to 9 were considered reflecting ERP components of interest, and were analyzed. We did not analyze subsequent factors further, as F10–F12 lacked a clear functional interpretation or showed no modulation by our experimental variables, while F13 extended beyond the analyzed time window. Table 6 reports the results of the models tested^3^.

**TABLE 6:** Results of the ANOVA models tested in the L1‐accent Temporal EFA.

	Df	MSE	*F*‐value	*η*_*p*_^2^	*p*
*F1 (N400)*					
Constraint	1, 41	2.58	145.58	0.780	< 0.001***
Face	1, 41	1.52	15.38	0.273	< 0.001***
Constraint*Face	1, 41	1.12	3.74	0.084	0.060
*F2 (P2)*					
Constraint	1, 41	1.21	19.57	0.323	< 0.001***
Face	1, 41	1.62	2.97	0.068	0.092
Constraint*Face	1, 41	1.59	0.45	0.011	0.507
*F3 (P2)*					
Constraint	1, 41	1.11	0.08	0.002	0.779
Face	1, 41	1.16	2.23	0.052	0.143
Constraint*Face	1, 41	1.50	0.02	< 0.001	0.878
*F4 (Slow wave)*					
Constraint	1, 41	2.99	25.31	0.382	< 0.001***
Face	1, 41	1.08	0.22	0.005	0.642
Constraint*Face	1, 41	0.71	0.01	< 0.001	0.937
*F5 (N400)*					
Constraint	1, 41	1.66	100.07	0.709	< 0.001***
Face	1, 41	1.49	2.21	0.051	0.145
Constraint*Face	1, 41	1.12	0.15	0.004	0.696
*F6 (P3b)*					
Constraint	1, 41	2.05	65.09	0.614	< 0.001***
Face	1, 41	1.52	6.73	0.141	0.013*
Constraint*Face	1, 41	0.94	8.19	0.166	0.007**
*F7 (P3b)*					
Constraint	1, 41	2.00	53.71	0.567	< 0.001***
Face	1, 41	1.14	4.42	0.097	0.042*
Constraint*Face	1, 41	1.50	7.41	0.153	0.009**
*F8 (Slow wave)*					
Constraint	1, 41	2.26	12.75	0.237	< 0.001***
Face	1, 41	0.87	0.65	0.016	0.426
Constraint*Face	1, 41	0.92	0.04	< 0.001	0.843
*F9 (N1)*					
Constraint	1, 41	1.00	0.11	0.003	0.740
Face	1, 41	1.67	2.66	0.061	0.110
Constraint*Face	1, 41	0.82	0.38	0.009	0.540

* *p*‐value < 0.05.

** *p*‐value < 0.01.

*** *p*‐value < 0.001.

##### 3.4.1.1 Early Components (N1/P2)

F9 showed a negative deflection peaking at 92 ms with a central distribution, consistent with the N1. It was not modulated by any condition. F2 and F3 showed a fronto‐central positive deflection peaking at 180 ms and 224 ms, respectively. These factors may reflect a temporal modulation of the P2 component, which was statistically divided into different factors. F2 exhibited a constraint effect, with larger fronto‐central positivity for unpredictable than predictable words (*b* = −0.751, *t*‐ratio_(41)_ = −4.424, *p* < 0.001), while F3 was not influenced by any of the experimental conditions.

##### 3.4.1.2 P3‐Like Responses

F6 and F7 peak at 276 and 332 ms, respectively, showing a posterior positivity for predictable words relative to unpredictable words (Figure 8), which may reflect a P3b response. The Constraint*Face interaction in F6 and F7 indicates that cueing the speaker identity is associated with a larger centro‐parietal positivity for predictable words (F6: *b* = 0.922, t‐ratio_(41)_ = 3.747, *p =* 0.002; F7: *b* = 0.860, *t*‐ratio_(41)_ = 3.372, *p =* 0.007) but not for unpredictable words (F6: *b* = 0.064, *t*‐ratio_(41)_ = 0.270, *p >* 0.999; F7: *b* = −0.169, *t*‐ratio_(41)_ = −0.685, *p >* 0.999).

##### 3.4.1.3 N400

F1 and F5 peak at 388 ms and 496 ms, respectively, showing a centro‐parietal negativity for unpredictable words relative to predictable words (Figure 8), a pattern that is consistent with the N400. Both factors exhibit a constraint effect, while F1 also showed a face effect, with reduced negativity when the speaker's identity was cued compared to when it was not cued (*b* = 0.747, *t*‐ratio_(41)_ = 3.922, *p* < 0.001). We also observed that both F1 and F5 present fronto‐central positive activity for predictable words, alongside the posterior negative activity observed for unpredictable words (Figure 7). This pattern may reflect distinct ERP components with different spatial distributions, complicating the interpretation of potential N400 amplitude modulations.

##### 3.4.1.4 Slow Waves

Among the factors emerging after the N400 time window (300–500 ms), F4 is associated with a centro‐parietal negativity for unpredictable words relative to predictable words (*b* = 1.34, *t*‐ratio_(41)_ = 5.031, *p* < 0.001). F8 shows a later fronto‐central positivity for unpredictable words relative to predictable words (*b* = −0.828, *t*‐ratio_(41)_ = −3.571, *p* < 0.001). None of these factors seems to be modulated by the face cue.

#### 3.4.2 L2‐Accent Temporal EFA

In the L2‐accent condition, we extracted a 23‐factor solution explaining 96% of the total variance. The unstandardized factor loadings for all extracted factors are reported in the Supporting Information.

**FIGURE 9:**
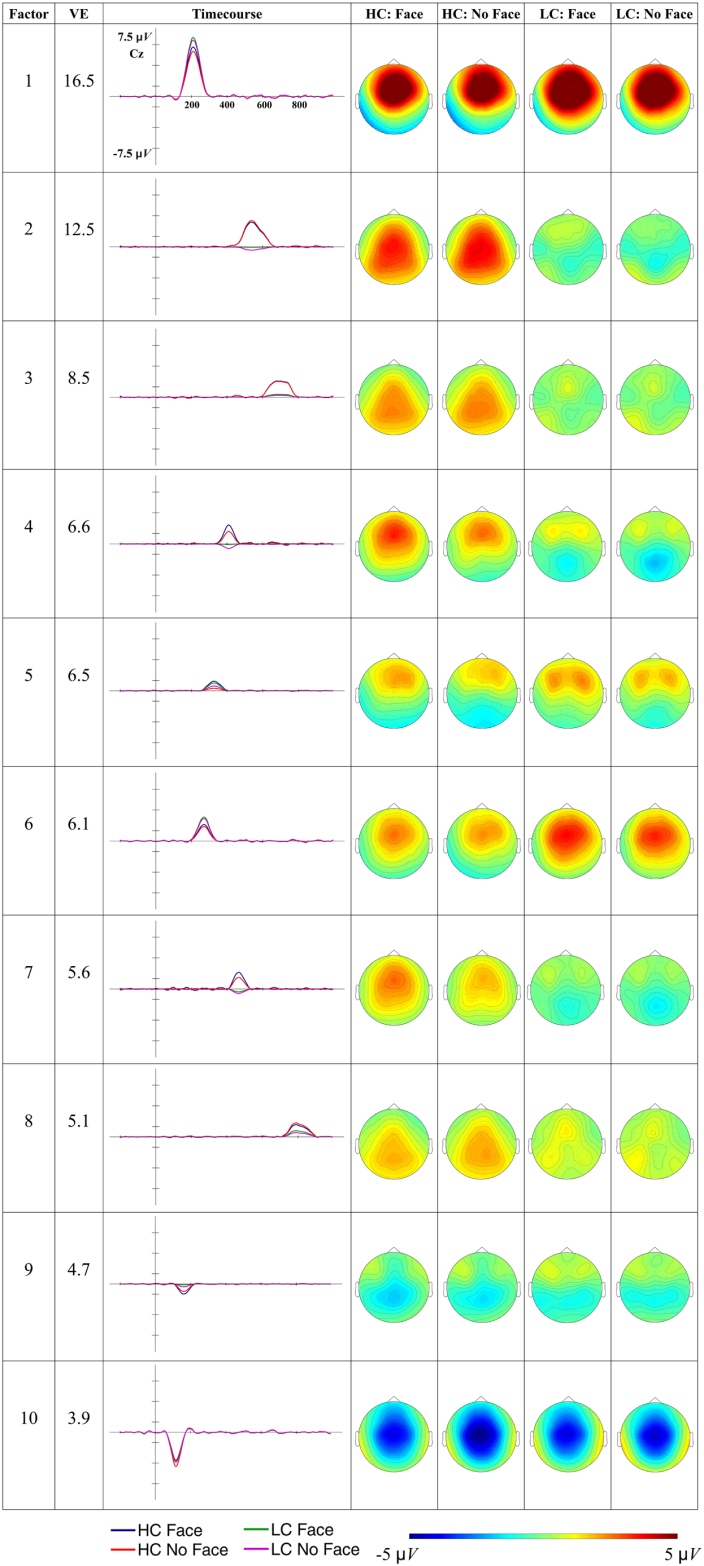
Time course and scalp distribution of the ERPs reconstructed factors from the L2‐accent Temporal EFA in the different experimental conditions. VE: percentage of total variance explained by the extracted factor. The reconstructed ERP time course at the electrode Cz and the scalp distribution of the peak amplitude (factor scores multiplied with the peak factor loading) are reported.

The factors explaining more than 3% of the total variance are shown in Figure 9.

**FIGURE 10:**
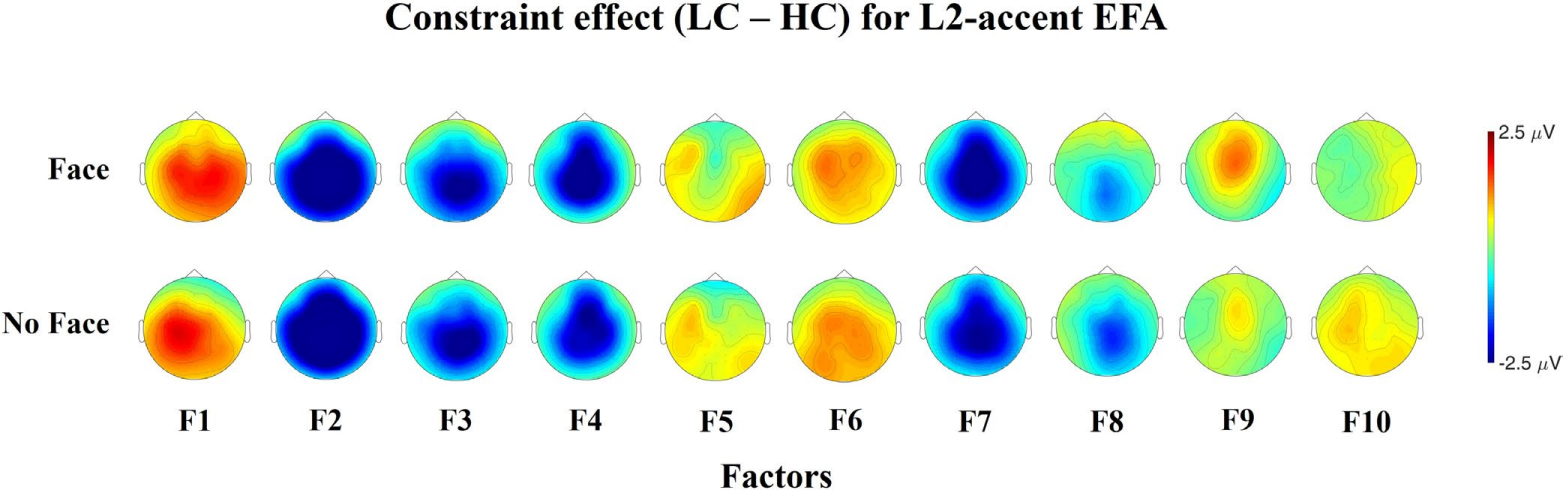
Topographic maps of the Constraint effect for factors explaining more than 3% of variance in the L2‐accent Temporal EFA.

Figure 10 presents the difference topographic maps for the Constraint effect in the factors explaining more than 3% of variance.

Factors from 1 to 10 were considered reflecting ERP components of interest, and were analyzed. Table 7 reports the results of the models tested.

**TABLE 7:** Results of the ANOVA models tested in the L2‐accent Temporal EFA.

	Df	MSE	*F*‐value	*η*_*p*_^2^	*p*
*F1 (P2)*					
Constraint	1, 41	2.66	22.63	0.356	< 0.001***
Face	1, 41	1.51	3.35	0.076	0.074
Constraint*Face	1, 41	1.86	0.09	0.002	0.760
*F2 (Slow wave)*					
Constraint	1, 41	4.35	148.95	0.784	< 0.001***
Face	1, 41	2.06	0.00	< 0.001	0.991
Constraint*Face	1, 41	1.41	3.76	0.084	0.059
*F3 (Slow wave)*					
Constraint	1, 41	2.26	91.20	0.690	< 0.001***
Face	1, 41	0.83	0.02	< 0.001	0.901
Constraint*Face	1, 41	0.88	0.09	0.002	0.770
*F4 (N400)*					
Constraint	1, 41	4.06	56.96	0.581	< 0.001***
Face	1, 41	1.85	11.39	0.217	0.002**
Constraint*Face	1, 41	0.82	0.88	0.021	0.353
*F5 (P3a)*					
Constraint	1, 41	2.51	0.71	0.017	0.405
Face	1, 41	1.16	9.18	0.183	0.004**
Constraint*Face	1, 41	1.35	0.29	0.007	0.590
*F6 (P3a)*					
Constraint	1, 41	1.64	25.78	0.386	< 0.001***
Face	1, 41	0.89	3.18	0.072	0.082
Constraint*Face	1, 41	1.07	0.14	0.003	0.710
*F7 (N400)*					
Constraint	1, 41	2.61	93.03	0.694	< 0.001***
Face	1, 41	1.33	5.90	0.126	0.020*
Constraint*Face	1, 41	1.33	0.69	0.016	0.413
*F8 (Slow wave)*					
Constraint	1, 41	1.67	30.24	0.424	< 0.001***
Face	1, 41	1.02	0.01	< 0.001	0.932
Constraint*Face	1, 41	1.40	1.59	0.037	0.214
*F9 (Early negativity)*					
Constraint	1, 41	1.36	16.97	0.293	< 0.001***
Face	1, 41	1.18	0.08	0.002	0.775
Constraint*Face	1, 41	1.45	2.64	0.060	0.112
*F10 (N1)*					
Constraint	1, 41	1.24	3.76	0.084	0.059
Face	1, 41	1.39	5.84	0.125	0.020*
Constraint*Face	1, 41	1.23	2.99	0.068	0.091

* *p*‐value < 0.05.

** *p*‐value < 0.01.

*** *p*‐value < 0.001.

##### 3.4.2.1 Early Components (N1/P2)

F10 presents a negative deflection peaking at 116 ms with a central distribution, consistent with the N1. This factor exhibits a face effect, showing reduced fronto‐central negativity when the speaker identity is cued compared to when it is not (*b* = 0.439, *t*‐ratio_(41)_ = 2.416, *p* = 0.02). F1 presents a positive deflection peaking at 208 ms with a fronto‐central distribution, consistent with the P2. This factor shows a constraint effect, with larger positivity for unpredictable than predictable words (*b* = −1.2, *t*‐ratio_(41)_ = −4.757, *p* < 0.001).

##### 3.4.2.2 P3‐Like Responses

F6 and F5 show fronto‐central positive deflections peaking at 272 ms and 324 ms, respectively, possibly reflecting a temporal modulation of the P3a response. F6 presents a constraint effect, with greater fronto‐central positivity for unpredictable words relative to predictable words (*b* = −1.0, *t*‐ratio_(41)_ = −5.078, *p* < 0.001). F5 presents a face effect, with increased fronto‐central positivity when the speaker identity is cued compared to when it is not (*b* = 0.502, *t*‐ratio_(41)_ = 3.030, *p* = 0.004).

##### 3.4.2.3 N400

F4 and F7 peak at 408 and 468 ms, respectively. Both factors seem to present a centro‐parietal negativity for unpredictable words relative to predictable words (Figure 10), a pattern that is consistent with the N400. Indeed, both factors show a constraint effect (F4: *b* = 2.35, *t*‐ratio_(41)_ = 7.547, *p* < 0.001; F7: *b* = 2.40, *t*‐ratio_(41)_ = 9.645, *p* < 0.001). Additionally, both factors show a face effect, with reduced centro‐parietal negativity when the speaker's identity is cued (F4: *b* = 0.708, *t*‐ratio_(41)_ = 3.375, *p* = 0.002; F7: *b* = 0.432, *t*‐ratio_(41)_ = 2.429, *p* = 0.02). As in the L1‐accent condition, both candidate factors for the N400 exhibit fronto‐central positive activity for predictable words, alongside the posterior negative activity observed for unpredictable words (Figure 9).

##### 3.4.2.4 Slow Waves

F2, F3, and F8 emerge after the N400‐time window (300–500 ms) and present a centro‐parietal negativity for unpredictable words relative to predictable words (F2: *b* = 3.93, *t*‐ratio_(41)_ = 12.205, *p* < 0.001; F3: *b* = 2.22, *t*‐ratio_(41)_ = 9.55, *p* < 0.001; F8: *b* = 1.1, t‐ratio_(41)_ = 5.499, *p* < 0.001).

## 4 Discussion

In the current study, we hypothesized that cueing the speaker's identity should facilitate the implementation of phonological predictions. Our main analysis supports this hypothesis, showing that cueing the speaker's identity—hence the speaker‐specific phonology of the upcoming word—is associated with a smaller N400 for predictable words but not for unpredictable words, and thus a larger predictability effect. However, this straightforward conclusion is complicated by the presence of positive deflections for predictable words within the N400 time window.

The presence of positive deflections within the analyzed time window may be due to the task used in the current study, in which participants were asked whether they expected the target word pronounced by the speaker in 25% of the trials. Brothers et al. (2015) used a similar task in every trial and showed that predicted targets compared to not predicted targets were associated with a reduction of the N250 amplitude. The authors interpreted this effect as a facilitation of visual word‐form processing due to word‐form prediction. Alternatively, Nieuwland (2019) proposed interpreting this effect as a modulation of the P3b component, given that in the considered N250 time window a strong positive deflection to predicted words was found.

It is generally accepted that a distinction can be made between two subcomponents of the P300 response. The P3a (or novelty P3) is a positive deflection with a fronto‐central distribution typically elicited in the oddball paradigm by infrequent non‐target stimuli. The P3b (or target P3) is characterized by a centro‐parietal distribution and is elicited by task‐relevant or target stimuli. In studies of language processing, the P3b has been observed in response to collocations (Molinaro and Carreiras 2010), idioms (Vespignani et al. 2010), antinomies (Roehm et al. 2007), and expressions featuring predictable verbs in acceptability judgment tasks (Freunberger and Roehm 2016). Its amplitude is thought to reflect the degree of correspondence between an internal representation and an external sensory stimulus during event categorization, with a larger amplitude reflecting a greater match (Kok 2001). Predicted words in a prediction task could elicit a rapid and strong P3b response. In our study, when speaker identity was cued, predictable words may have elicited a greater P3b response, reflecting a greater degree of correspondence between the internal representation of the upcoming word and the external input due to phonological prediction.

The results of the Temporal EFAs (Dien 2012; Dien and Frishkoff 2005; Scharf et al. 2022) provide evidence consistent with this hypothesis. In the L1‐accent condition, predictable words elicit a centro‐parietal positivity compared to unpredictable words, possibly reflecting a P3b response. Moreover, this P3b response for predictable words was more pronounced when speaker identity was cued compared to when it was not. The selectivity of the face cue effect for predictable words suggests that the enhanced P3b response does not simply reflect the matching between the face cue and the speaker's voice. As observed by Nieuwland (2019), it remains unclear whether the P3b response elicited by a task requiring an explicit decision on linguistic stimuli reflects recognition, word‐form analysis, or decision‐related processes occurring after stimulus recognition. Our data provide some insights into this issue. In our task, participants categorized the final word of each sentence as predictable or not, regardless of how it was pronounced. While predictable words were inherently task‐relevant, the presence of the face cue was not critical for deciding whether a stimulus was expected. Therefore, the observed modulation of the P3b in response to the face cue likely reflects facilitation in recognition or word‐form analysis rather than a decision‐making process.

In the L2‐accented condition, the face cue effect appears as a modulation of the N1 and the P3a response. Cueing the speaker's identity was associated with a suppression of the N1 response, regardless of sentence constraint. The N1 component is thought to reflect basic operations involved in constructing perceptual representations (Näätänen and Picton 1987). It has been suggested that the N1 signals the detection of acoustic changes in the environment (Hyde 1997), with its amplitude increasing in response to heightened attention to stimuli (Hillyard et al. 1973; Knight et al. 1981; Mangun 1995; Ritter et al. 1988). In our study, the suppression of the N1 observed for L2‐accented words may reflect the system's uncertainty in mapping the speech signal onto linguistic categories. Maintaining uncertainty about a category may be beneficial, as additional information becomes available as speech unfolds. Evidence suggests that later portions of a spoken stimulus can influence the interpretation of earlier portions (i.e., right‐context effects in word recognition; Bard et al. 1988; Connine et al. 1991; Dahan 2010; Grosjean 1985), suggesting that listeners retain some degree of uncertainty about the speech signal for a limited time. When the speaker identity is cued, listeners may delay the categorization of the first phoneme of the target word, where L2‐accented speech diverges from the standard phonology, awaiting further information that could guide interpretation.

Additionally, L2‐accented words seem to elicit a P3a rather than a P3b response. The P3a has been linked to processes related to the orienting response (Cycowicz and Friedman 1997; Friedman et al. 1998; Knight and Nakada 1998). These processes do not simply reflect the detection of a deviant or novel event. Rather, they occur after the brain has identified the deviation, including bringing it to conscious awareness for further evaluation and determining an appropriate response. In our study, participants' limited familiarity with L2‐accented speech may have elicited a P3a response. An early P3a modulation was elicited by word predictability, followed by a later modulation associated with the face cue. The earlier modulation of P3a may reflect an increased resource allocation for unpredictable words compared to predictable words. The later modulation of the P3a may be associated with a delay in the initial categorization of the speech signal when the speaker identity is cued, with the system allocating more resources to extract information that facilitates phonetic categorization at word onset.

In both accent conditions, we observed a sustained centro‐parietal negativity for unpredictable words compared to predictable words, extending beyond the canonical N400 time window. Sentence‐final negativities emerge primarily when a decision about the linguistic stimuli is required, suggesting that they may be related to maintaining information relevant to the task (Stowe et al. 2018). This aligns with ERP studies showing slow negative waves during tasks that involved activation of working memory (Lang et al. 1987; McCallum et al. 1988; Peronnet and Farah 1989; Ruchkin et al. 1988, 1995). In low‐constraining sentence contexts, where predictions about upcoming words are weaker or more uncertain, the system may maintain multiple potential candidates in working memory. As a result, the decision‐making process for the target word becomes more complex compared to highly constraining contexts, where one lexical candidate is far more likely than any alternative, leading to a prolonged negativity for unpredictable words.

Finally, we observed a late fronto‐central positivity for unpredictable words compared to predictable words in the L1‐ but not in the L2‐accented condition. Late frontal positivities can be elicited by semantically congruent sentence completions and are usually more pronounced for unpredictable words than for predictable words (Van Petten and Luka 2012). It has been proposed that late frontal positivities reflect disconfirmed lexical predictions rather than general conceptual predictions (Thornhill and Van Petten 2012; Van Petten and Luka 2012). In the L1‐accent condition, predictable words likely matched the expected lexical form, whereas unpredictable words did not. For L2‐accented words, their non‐standard phonology may have hindered lexical access, which, in turn, made the comparison between expected lexical form and pronounced word more challenging.

### 4.1 What Do Our Data Say About Linguistic Prediction Models?

The results of our study provide compelling evidence supporting the involvement of phonological representations in prediction, at least in highly informative and constraining contexts. The analysis of the observed amplitude in the N400 time window suggests that cueing speaker identity is associated with easier processing of predictable words due to the pre‐activation of the phonological word form. Temporal EFA revealed substantial differences between the L1‐ and L2‐accented conditions in the processes by which comprehenders use available information to predict upcoming speech. In the L1‐accent condition, the face‐cueing effect for predictable words appears to be driven by a larger P3b response, reflecting a greater degree of correspondence between the internal representation of the upcoming word and the external input. In the L2‐accented condition, we did not find a clear factor associated with a face‐cueing effect for predictable words. Instead, we observed that cueing speaker identity is associated with a smaller N1 and a larger P3a, suggesting that comprehenders, when expecting non‐standard pronunciations, adopt a more flexible processing strategy—maintaining uncertainty about the speech signal while leveraging contextual information to facilitate comprehension.

Our findings help elucidate the mechanisms underlying phonological predictions. A standard assumption in the psycholinguistic literature is that activation of a lexical item spreads to similar or related items, due either to learned association in semantic networks (Anderson 1983; Collins and Loftus 1975; Hutchison 2003) or overlapping semantic features between concepts (McRae et al. 1997). More recent cognitive frameworks postulate the presence of recurrent interactions between different levels of representation. As a result, the activation of a semantic representation could percolate top‐down, influencing the processing of the phonologic (Huettig et al. 2022) or orthographic (Kim and Lai 2012; Molinaro et al. 2013) word features. These accounts extend beyond word‐to‐word semantic priming to encompass a broader sense of priming, which includes facilitation based on both linguistic and non‐linguistic contextual information, considering the pre‐activation of low‐level features as a downstream consequence of the (pre‐)activation of lexical‐semantic representations. In our experimental paradigm, the speaker's face serves as a cue to the phonological properties of the upcoming word before its presentation. Although spreading of activation accounts acknowledge that non‐linguistic context can influence phonological predictions by making the pre‐activation of lexical‐semantic representations more or less focal, they fail to explain how a phonologically informative non‐linguistic cue can modulate the pre‐activation of phonological representations.

Prediction‐by‐production accounts represent a valuable framework for understanding how listeners use the available information to generate phonological predictions. According to these models, prediction during comprehension relies not only on associative mechanisms but also on processes traditionally attributed to language production (Huettig 2015; Pickering and Gambi 2018; Pickering and Garrod 2007, 2013). Neurophysiology studies comparing the processing of high‐ and low‐constraining sentence frames followed by a picture to be produced or by a word to be perceived showed very similar brain time‐frequency modulations between planning in word production and prediction during comprehension (Gastaldon et al. 2020) and that such neural markers are altered when speech planning is impaired (Gastaldon et al. 2023). These findings suggest that the language production network contributes to some extent to the prediction of the last word in high‐constraint sentences. Although prediction‐by‐production accounts are not entirely in agreement regarding the processes and representations involved in linguistic prediction (for a review, see Gastaldon et al. 2024), they often assume a role of event simulation in the generation of predictions.

Pickering and Garrod (2013) proposed that listeners covertly imitate the unfolding utterance in order to derive an internal representation of the speaker's percept, which constrains the prediction of upcoming speech. In this account, predictions are generated through forward models of the upcoming speech in the form of “impoverished” production representations. Later accounts have diverged from Pickering and Garrod (2013), arguing that prediction relies on fully fledged production representations (Huettig 2015; Pickering and Gambi 2018) and that event simulation is a separate mechanism interacting with production (Huettig 2015). Despite the different status of event simulation in prediction‐by‐production accounts, this mechanism could help to explain how listeners use the available information to generate phonological predictions, especially for the L1‐accented speaker. The accuracy of an internal model of the speaker should increase with the comprehender's similarity to the speaker (Pickering and Garrod 2013). The internal model may incorporate different types of speaker‐related information, such as shared background knowledge (Pickering and Gambi 2018) and phonological features, allowing for the prediction of how the speaker will pronounce the upcoming word. In our paradigm, comprehenders likely relied more on simulation with the L1‐accented speaker, as they could construct a more accurate internal model of the speaker.

The literature includes other prediction models that could account for the implementation of phonological predictions without relying on event simulation or word production mechanisms. Kuperberg and Jaeger introduced a multi‐representational hierarchical generative model (Kuperberg 2016; Kuperberg and Jaeger 2016) in which comprehenders rely upon internal generative models—defined as a set of hierarchically organized internal representations—to probabilistically pre‐activate information at multiple levels of representation. The pre‐activation of linguistic representations maximizes the probability of accurately recognizing the incoming information. Internal representations are derived from a limited number of observations, encompassing both linguistic and non‐linguistic information, and they may also include knowledge of the speaker's sound structure (Connine et al. 1991; Szostak and Pitt 2013). Listeners may learn different generative models corresponding to different statistical environments (Kleinschmidt and Jaeger 2015), and these models will likely be more precise with increasing familiarity with the speaker.

In a previous behavioral study (Sala et al. 2024), we used a similar experimental design to investigate whether speaker identity (L1‐ vs. L2‐accented) is used to implement phonological predictions. Participants were required to perform a lexical decision task on the spoken target word. In the case of L2 accent, participants were explicitly asked to accept mispronounced words as real. The results showed that cueing the speaker's identity speeded up the RTs for predictable words, regardless of the speaker's accent, suggesting that phonological predictions were similarly instantiated for both the L1‐ and the L2‐accented speaker. In the present study, by tapping into electrophysiological responses, we found clearer evidence of phonological predictions for the L1‐accented speaker. This apparent discrepancy in the results can be explained by the different tasks used. In the behavioral study, participants were required to discriminate between real words mispronounced by the L2‐accented speaker and non‐words, whereas in the present study, participants were asked to indicate whether they expected the spoken word regardless of its pronunciation. Participants may have implemented phonological predictions more flexibly in the lexical decision task, as such predictions could be useful in distinguishing between mispronounced real words and non‐words pronounced by the L2‐accented speaker. This aligns with Kuperberg and Jaeger's (2016) proposal that the degree and level of predictive pre‐activation depend on its expected utility, which is shaped by comprehenders' goals and their assessment of the reliability of prior knowledge and bottom‐up input.

To conclude, comprehenders seem to consider the phonological variability between speakers in predicting the upcoming speech. These findings shed light on both the level(s) of representation and the processes involved in generating predictions. Our results support the notion that linguistic prediction involves the pre‐activation of phonological representations. Furthermore, they also suggest that phonological predictions do not derive solely from the passive spreading of activation between higher‐level representations that percolate to phonological representations. Rather, they may be influenced by other mechanisms that are more sensitive to the phonological characteristics of the speaker, such as event simulation or internal generative models. The predictive nature of the task represents a potential limitation of the present study, as it may have influenced participants' reliance on phonological predictions. Moreover, the absence of the three‐way interaction between constraint, accent, and face in our main analysis should be taken cautiously, also considering that differences in the face‐cueing effect across accent conditions did emerge in the Temporal EFA analysis, where simpler statistical models were tested separately for each accent condition. Finally, it would be crucial to investigate whether and how phonological predictions are implemented in natural conversations, where additional communicative goals and constraints may shape predictive processes. This would provide a more comprehensive understanding of the flexibility of the perceptual system to predict the upcoming speech.

## Author Contributions

**Marco Sala:** conceptualization, investigation, data curation, formal analysis, visualization, writing – original draft. **Francesco Vespignani:** conceptualization, writing – review and editing. **Simone Gastaldon:** conceptualization, writing – review and editing. **Laura Casalino:** investigation, writing – review and editing. **Francesca Peressotti:** conceptualization, supervision, funding acquisition, writing – review and editing.

## Ethics Statement

The research adhered to the principles outlined in the Declaration of Helsinki. The research protocol was approved by the Ethics Committee for Psychological Research of the University of Padova (protocol number: 5181). Participants provided their informed consent before participating in the experiment. They also consented to the use of their data (collected anonymously) for research aims.

## Conflicts of Interest

The authors declare no conflicts of interest.

## Supporting information


**Data S1:** psyp70135‐sup‐0001‐Supinfo01.docx.

## Data Availability

The data that support the findings of this study are openly available in Open Science Framework at https://osf.io/brvkx/.

## Endnotes

^1^
Recent work in psycholinguistics has questioned the use of the terms “native speaker” and “non‐native speaker” (Cheng et al. 2021; Weissler et al. 2023). We use the terms *L1* and *L2 accent* (or speaker) instead, as suggested by one of the reviewers.

^2^
In the ERP literature both PCA (Principal Component Analysis, see Dien and Frishkoff 2005; Dien 2012) and EFA (Exploratory Factor Analysis, see Scharf et al. 2022) have been used to estimate the underlying components of the ERP data. However, the differences between PCA and EFA estimates are negligible for ERP data due to the high correlations between sampling points and to the high number of variables (Dien and Frishkoff 2005; Scharf and Nestler 2019; Widaman 1990, 2007).

^3^
As suggested by a reviewer, we added in parenthesis a putative label of the ERPs component that could be related to each specific factor. We underline that these interpretative links are based on a qualitative inspection of the latency and topography of the factors.
