# Supplementary material for: In the Words of Others: ERP Evidence of Speaker‐Specific Phonological Prediction
Source: Psychophysiology. 2025 Sep 1;62(9):e70135. doi: 10.1111/psyp.70135 (PMC12402685; doi:10.1111/psyp.70135)

**Supplementary materials**

*Behavioral data analysis*

Responses to the written questions could be scored either 0 to 1, which respectively indicated “not expected” and “expected” answers. Prediction accuracy was analyzed using generalized linear mixed-effects models (binomial distribution with logit link). Model comparison was conducted using a hierarchical model comparison approach as in the analysis of the ERP data.

Table 1 reports the results of the model comparison for prediction accuracy.

**Table 1.** The comparison of GLMER models. Deviance = residual deviance; dAIC = difference between AIC of each model and the model with lower AIC; AICw = AIC weight.

| **Models** | **Deviance** | **dAIC** | **AICw** |
| --- | --- | --- | --- |
| M0. Prediction accuracy ~ (Constraint\|Participant) + (1\|Item) | 1655.6442 | 110.19 | 0.0 |
| M1. Prediction accuracy ~ Accent + (Constraint\|Participant) + (1\|Item) | 1654.8073 | 111.35 | 0.0 |
| **M2. Prediction accuracy ~ Accent + Constraint + (Constraint\|Participant) + (1\|Item)** | **1541.4582** | **0.0** | **0.51** |
| M3. Prediction accuracy ~ Accent + Constraint + Face + (Constraint\|Participant) + (1\|Item) | 1541.4503 | 1.99 | 0.19 |
| M4. Prediction accuracy ~ Accent + Constraint + Face + Constraint*Accent + (Constraint\|Participant) + (1\|Item) | 1539.8934 | 2.44 | 0.15 |
| M5. Prediction accuracy ~ Accent + Constraint + Face + Constraint*Accent + Constraint*Face + (Constraint\|Participant) + (1\|Item) | 1538.6404 | 3.18 | 0.10 |
| M6. Prediction accuracy ~ Accent + Constraint + Face + Constraint*Accent + Constraint*Face + Accent*Face + (Constraint\|Participant) + (1\|Item) | 1538.5292 | 5.07 | 0.04 |
| M7. Prediction accuracy ~ Accent + Constraint + Face + Constraint*Accent + Constraint*Face + Accent*Face + Accent*Constraint*Face + (Constraint\|Participant) + (1\|Item) | 1538.4802 | 7.02 | 0.02 |

As shown in Table 1, model comparison indicates that the best-fitting model (lower delta AIC and higher AIC weight) for prediction accuracy is Model 2:

*Prediction accuracy ~ Accent + Constraint + (Constraint|Participant) + (1|Item)*

The results of the best-fitting model for prediction accuracy (Model 2) are reported in Table 2. The effect of Constraint indicates that target words were more likely to be categorized as “expected” in the High Constraint than in the Low Constraint condition.

**Table 2.** Results of the best-fitting GLMER model.

|  | **Estimate** | **CI (95%)** | **Std. Error** | ***z-value*** | ***p-value*** |
| --- | --- | --- | --- | --- | --- |
| Intercept | -0.045 | [-0.405 0.315] | 0.184 | -0.245 | .807 |
| Accent[Foreign] | -0.067 | [-0.211 0.077] | 0.074 | -0.915 | .360 |
| Constraint[HC] | 3.354 | [3.000 3.709] | 0.181 | 18.511 | < .001 |

*L1-accent Temporal EFA*

Figure 1 illustrates the unstandardized factor loadings for the L1-accent condition.

**Figure 1.** Unstandardized Factor Loadings after Geomin Rotation in the L1-accent condition EFA. Each colored line represents the factor loadings of a factor. Higher factor loadings imply that the factor contributes more to the voltage at a sampling point. The factors are numbered by the amount of variance they explain.


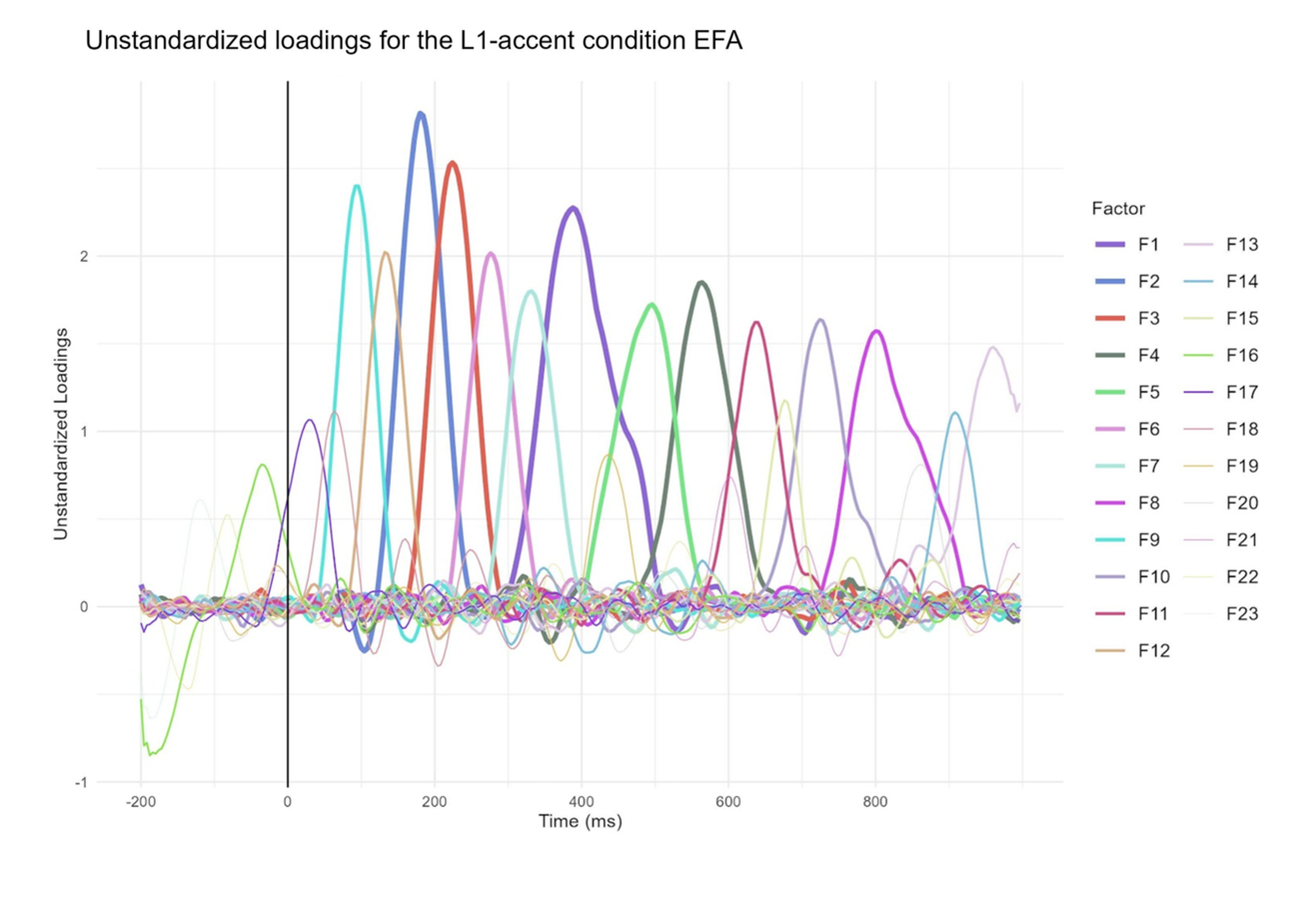


*L2-accent Temporal EFA*

Figure 2 illustrates the unstandardized factor loadings for the L2-accent condition.

**Figure 2.** Unstandardized Factor Loadings after Geomin Rotation in the L2-accent condition EFA. Each colored line represents the factor loadings of a factor. Higher factor loadings imply that the factor contributes more to the voltage at a sampling point. The factors are numbered by the amount of variance they explain.


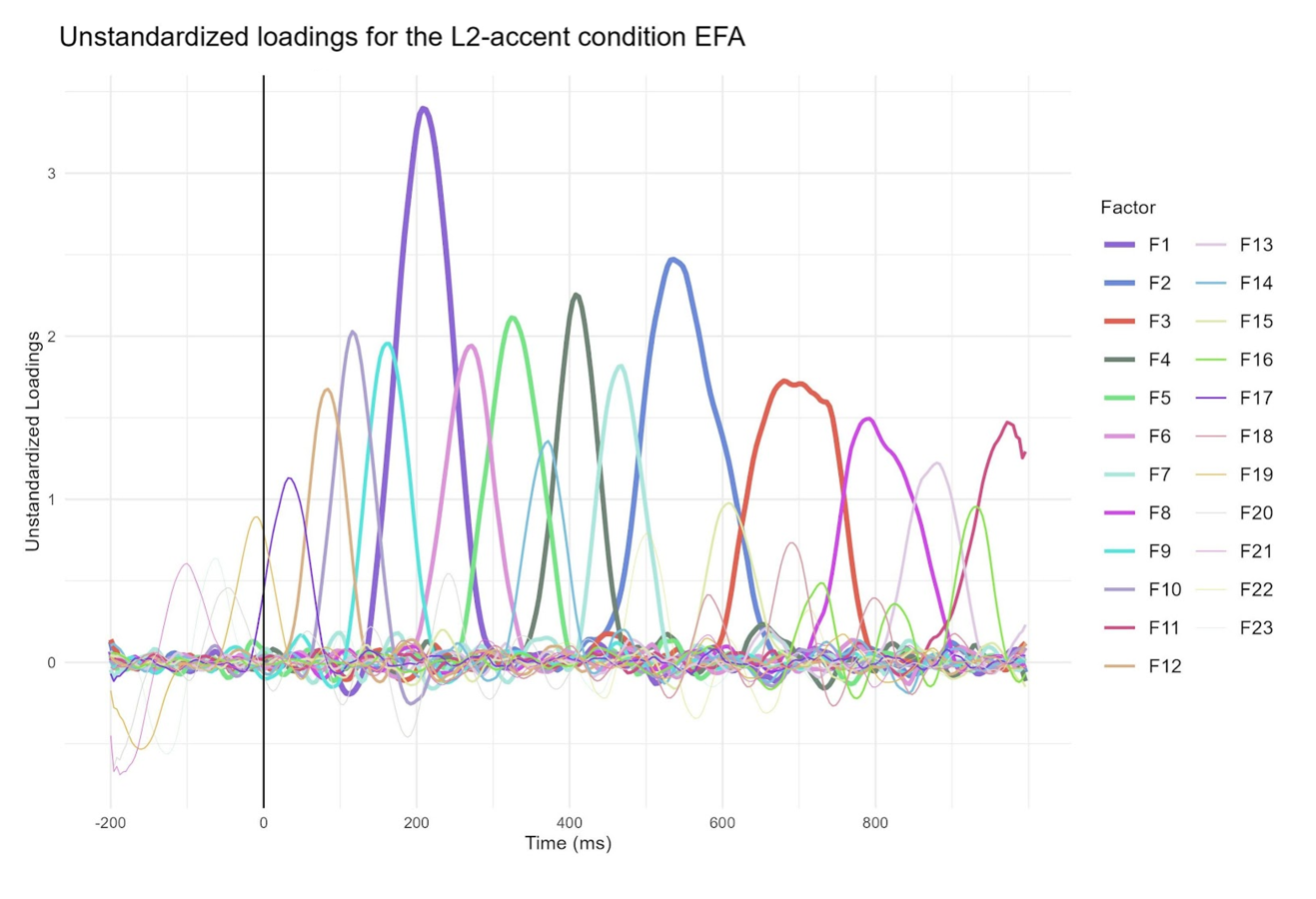

Supplement: Supplementary file 1 — Data S1: psyp70135‐sup‐0001‐Supinfo01.docx. [file PSYP-62-e70135-s001.docx]
